# A Deep Learning Framework for Processing and Classification of Hyperspectral Rice Seed Images Grown under High Day and Night Temperatures

**DOI:** 10.3390/s23094370

**Published:** 2023-04-28

**Authors:** Víctor Díaz-Martínez, Jairo Orozco-Sandoval, Vidya Manian, Balpreet K. Dhatt, Harkamal Walia

**Affiliations:** 1 University of Puerto Rico, Mayagüez, PR 00681, USA; victor.diaz16@upr.edu (V.D.-M.); jairo.orozco@upr.edu (J.O.-S.); 2 University of Nebraska-Lincoln, Lincoln, NE 68588, USA; balpreetkaur.dhatt@bayer.com (B.K.D.); hwalia2@unl.edu (H.W.)

**Keywords:** hyperspectral images, 3D-convolutional neural networks, deep neural networks, rice seeds, graphical user interface, high day and night temperatures

## Abstract

A framework combining two powerful tools of hyperspectral imaging and deep learning for the processing and classification of hyperspectral images (HSI) of rice seeds is presented. A seed-based approach that trains a three-dimensional convolutional neural network (3D-CNN) using the full seed spectral hypercube for classifying the seed images from high day and high night temperatures, both including a control group, is developed. A pixel-based seed classification approach is implemented using a deep neural network (DNN). The seed and pixel-based deep learning architectures are validated and tested using hyperspectral images from five different rice seed treatments with six different high temperature exposure durations during day, night, and both day and night. A stand-alone application with Graphical User Interfaces (GUI) for calibrating, preprocessing, and classification of hyperspectral rice seed images is presented. The software application can be used for training two deep learning architectures for the classification of any type of hyperspectral seed images. The average overall classification accuracy of 91.33% and 89.50% is obtained for seed-based classification using 3D-CNN for five different treatments at each exposure duration and six different high temperature exposure durations for each treatment, respectively. The DNN gives an average accuracy of 94.83% and 91% for five different treatments at each exposure duration and six different high temperature exposure durations for each treatment, respectively. The accuracies obtained are higher than those presented in the literature for hyperspectral rice seed image classification. The HSI analysis presented here is on the Kitaake cultivar, which can be extended to study the temperature tolerance of other rice cultivars.

## 1. Introduction

Rice is a food staple for more than 3.5 billion people around the world, particularly in Asia, Latin America, and parts of Africa. According to the International Grains Council (IGC), the total volume of milled rice produced worldwide reached 504 million metric tons in the 2021/2022 crop year. There is a demand for high-quality rice which is increasing worldwide. Rice quality-related genes and their role in regulating structure and components for improving quality of rice are being studied [1]. However, the global temperature is predicted to rise by 0.3 to 4.8 deg C. This supra-optimal temperature cause changes in the plants in the morphological, physiological, biochemical, as well as gene level alterations. High temperature stress affects rice seed growth in several ways. It results in reduced grain quality and crop yield. It affects the grain size, color, and phenotypic characteristics. Variation in grain width and height for mature grain under high night temperature stress is reported in [2]. Phytohormones such as auxin and cytokinin are affected by high temperatures affecting the starch filling in the grain adversely and causing chalkiness, changing low quality grains and lowering the market value.

Global mean day and night temperatures are rising throughout the world, affecting cereal and grain cultivars. Hence, it becomes increasingly important to develop an automatic mechanism to monitor the quality of grains and cereals grown under high temperatures for market acceptance as well as to study its implications on consumer health. It is essential to utilize imaging technology and to analyze the resultant images to determine phenotypic variations in grains and crops that feed the communities. 

Hyperspectral images (HSIs) record the spectral signature of materials imaged in highly sampled wavelengths called spectral bands. Each material has a unique spectral signature and can be used to distinguish it from other materials. Additionally, under environmental stressors, the spectral signatures of naturally occurring materials, food crops, water bodies, and minerals can be identified. Imaging techniques have been used for crop quality assessment and prediction. Deep Learning (DL) has become a fundamental branch of Artificial Intelligence (AI), due to its ability to discover patterns by mimicking the functioning of neurons in the human brain. The Deep Neural Networks (DNNs) have exceptional learning capability, and as a result, it has been used in different areas of Machine Learning (ML) and AI. The DNNs have been used in many applications such as health, signal processing, data analysis, and context of computing among others [3]. 

DL methods are also being applied to classify crop seed images. RGB images of maize seeds have been classified in [4] using a deep learning architecture. The size, shape and color of rice is used to identify weedy rice that are a problem to regular rice varieties. An RGB-based image analysis and machine learning approach is presented in [5] to identify weedy rice from regular rice seeds. An overview of spectroscopic imaging configuration, key wavelengths, spectral, and spatial resolutions for multispectral imaging for seed phenotyping and quality monitoring is discussed in [6]. In order to study the phenotypic variation in rice seeds due to environmental stressors, RGB color images and hyperspectral images are acquired and analyzed [7]. Four varieties of rice seeds have been classified using hyperspectral images in [8]. A comparison of milled and brown rice samples is carried out using NIR-HSI to detect rice varieties [9]. Another study reported corn seed viability using hyperspectral imaging. A wavelength range of 900–1700 nm is employed to obtain spectral images of three different varieties of naturally aged watermelon seed samples and to detect the viability of seeds using partial least square discriminant analysis (PLS-DA) model. Germination prediction of beet seeds using HSIs and machine learning approaches such as support vector machines, random forest, and gradient boosting classifiers is presented in [10]. 

Regularly grown rice seed varieties have been classified using Convolutional Neural Networks (CNNs) in [11,12]. All crop seeds have starch, fat, and enzymes and hence their spectral signatures overlap. A common drawback in a hyperspectral image-based classification of different crop seeds is the limited number of samples available for training a deep learning neural network. To overcome this limitation, a deep transfer learning neural network model is used in [13] for training a deep learning network to classify a particular variety of seed, and to reuse the network to classify other types of crop seeds. Most of the above publications are on crop seed growth under normal conditions. Due to the changing climate on the planet, spectroscopy is beginning to be used to understand the effects of high temperatures on grain quality. HSIs of rice seeds are from two classes: control and high day and night temperatures, which are classified using a 3 Dimensional Convolutional Neural Network (3D-CNN) architecture in [14]. Currently, there is no study evaluating the imaging spectroscopy for distinguishing seeds grown under different hours of exposure to higher day and/or night temperatures. The main contributions of this paper are the following:

(a) Classification of HSIs of rice seeds grown under different exposure durations to different high day and/or night temperature treatments using DL architectures;
(b) A DL framework for a comprehensive analysis of hyperspectral images of seeds. We present results from rice seeds grown under High Night Temperature (HNT), High Day and High Night Temperature (HDNT) stressors, and control environments. Our framework includes options for calibration, preprocessing, and segmentation for HSI seed image extraction from a panel image of seeds, spectral analysis, spatial–spectral feature extraction, as well as classification using two different DL neural network architectures: 3D-CNN and Deep Neural Network (DNN);
(c) A software application of the DL seed image processing framework is the first of its kind for processing crop seed HSIs.

The rest of the paper is organized as follows. Section 2 presents the materials and methods that describe the framework with various Graphical User Interfaces, Section 3 presents the results of rice seed HSI classification using both 3D-CNN and DNN, Section 4 presents a discussion, and Section 5 the conclusions.

## 2. Materials and Methods

**Figure 1:**
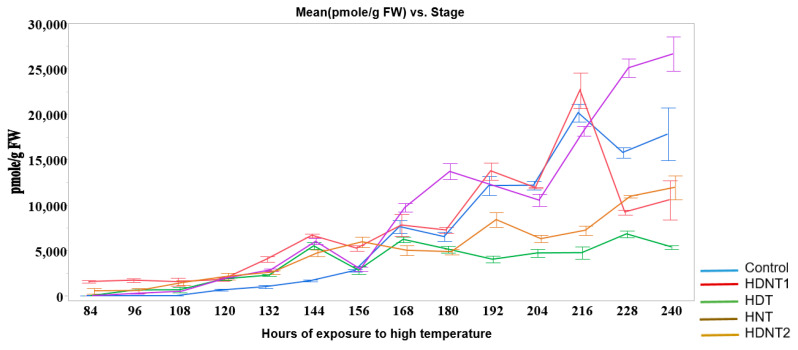
Auxin quantification at different hours of exposure of rice seeds to high temperatures.

This section describes the high temperature treatments that the rice plants have gone through. The rice (*Oryza sativa*) variety Kitaake (a temperate japonica cultivar) is used in this study. This section also presents the rice seed HSI calibration and visualization tools, and the deep learning architectures for the analysis and classification of the images. Rice plants are moved to the high-temperature area after fertilization. The temperatures for the four different treatments are shown in Figure 1. This figure also shows Auxin quantification in pmoles/g for the four different treatments and the control group during which the temperatures are normal. The new HDNT is referred to as HDNT2 treatment and is included in the image analysis. The Auxin level increases as the temperature increases. In order to identify phenotypic changes as temperature increases, we analyze the hyperspectral images of sample seeds from different hours of exposure to high temperatures. The images of the seeds are acquired at 168, 180, 204, 216, 228, and 240 h of exposure to high temperatures after the fertilization stage of the plants. Hence, the images analyzed are from five different treatments including the control, and from six different hours of exposure to high temperatures. 

### 2.1. Hyperspectral Rice Seed Datasets

The hyperspectral images of rice seeds grown under high day/night temperature environments and the control environment are taken with a high-performance line-scan image spectrograph (Micro-Hyperspec Imaging Sensors, Extended VNIR version) [1]. This sensor covers the spectral range from 600 to 1700 nm. A hyperspectral camera that records the spectrum from 597.21 nm to 1703.93 nm with an inter-band sampling of 4.14 nm is used to record the rice seed images. Each image has 268 bands. The images are recorded as hypercubes of the dimension 1250 × 450 × 268. Hypercubes are recorded for rice seeds grown in control, and high day and night temperature. Table 1 gives the details of the temperature treatments of the rice seeds and number of seed images acquired in each category.

### 2.2. Calibration and Segmentation of Rice Seed HSI

**Figure 2:**
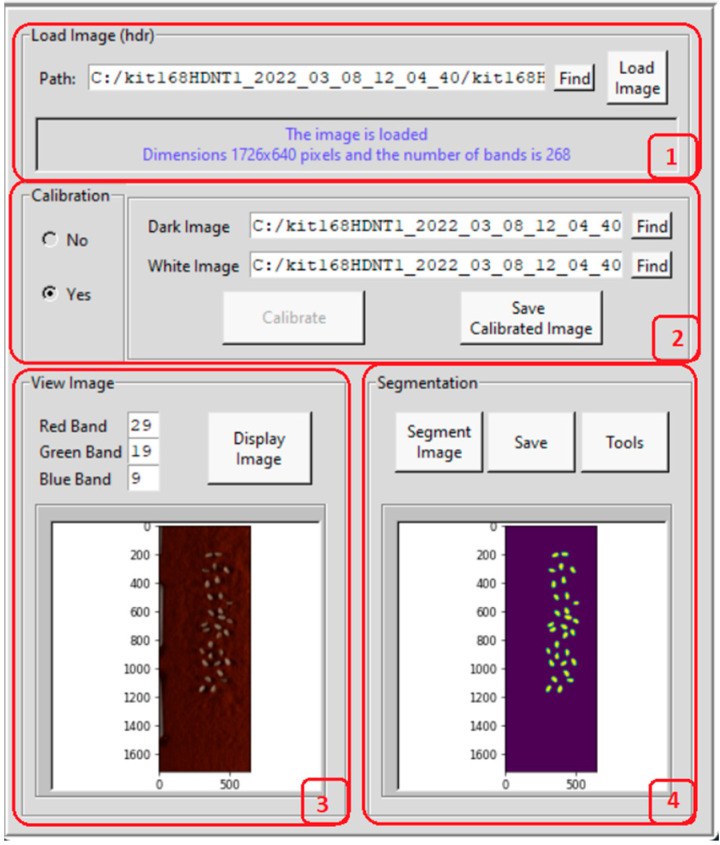
GUI for hyperspectral seeds image calibration and segmentation. There are 4 modules with functions: load image, save calibrated image, display RGB image and segment image. The seeds are visible in the image panels in modules 3 and 4.

**Figure 3:**
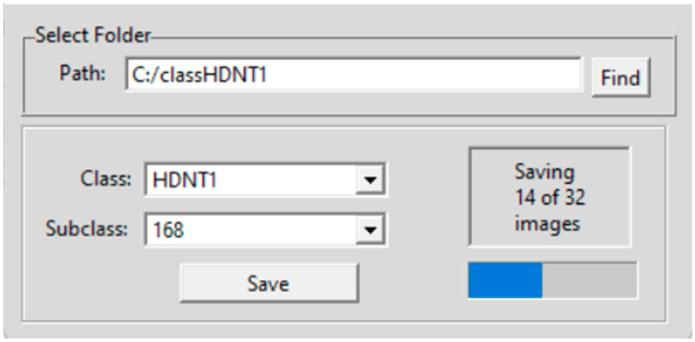
GUI for hyperspectral seed image extraction.

**Figure 4:**
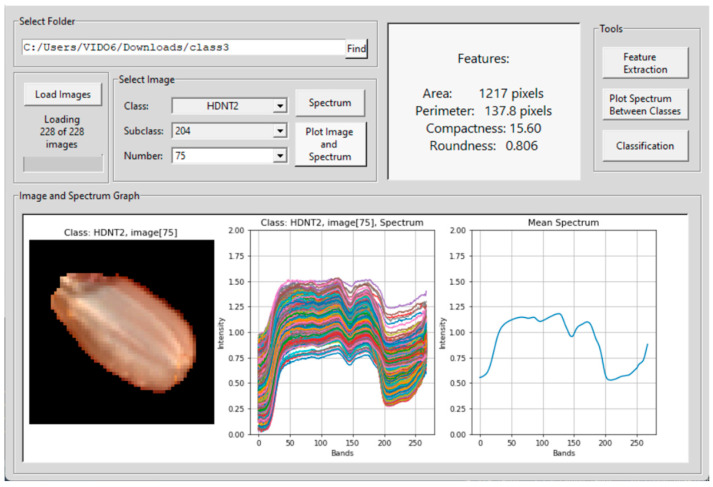
GUI for hyperspectral seed image spectrum and feature extraction. The colors indicate pixel vectors of sample pixels in the rice seed HSI, and the single blue spectrum is the mean pixel spectral vector of all the sample pixels.

We present the processing and DL classification framework of HSIs of rice seeds using Graphical User Interfaces (GUIs). Figure 2 presents the GUI for HSI rice seed calibration and segmentation. The calibration is conducted using the Shafer model [15] given by the equation,

(1) $$I_{c}\;=\;\frac{I-I_{d}}{I_{w}-I_{d}}$$

where *I_c_* is the calibrated constant reflection value at a predetermined wavelength, I is the original spectra in the form of intensity, *I_w_* is the white reference image captured from a white Teflon tile with 100% reflection, and *I_d_* is the dark reference image with 0% reflection obtained with the light source turned off and the camera lens covered with a cap. The module 1 in the GUI presents the dimension of the image hypercube that includes the number of rows, number of columns, and number of bands. Module 2 is used for calibration of the image. In this sub-window, the dark and white reference images are uploaded, a button is provided for calibration, and another for saving the calibrated image. In module 3, the user can select the red, green, and blue bands as any three out of the 268 bands for displaying the image as an RGB color composite. Module 4 is for segmenting the seeds from the background, which opens a new interface shown in Figure 3 for extracting each seed and saving the image. The "Find" button is used to select the path in which the individual rice seed images are to be saved. In this case, there are four different datasets of rice seed high temperature treatments, and six subclasses of high temperature exposure duration for each of the four treatment class. There is one control class of HSIs for normal temperature grown rice seeds. The individual seed images are saved in a folder for training the 3D-CNN architecture. The tools button in module 4 in Figure 2 opens another interface shown in Figure 4. In the left panel of this module, each individual band can be visualized as an image. In the select image panel, an image from a temperature treatment and a subclass of the number of hours of exposure and band number can be input for the image to be displayed in the bottom left panel. For example, band number 75 from a HDNT2 rice seed HSI, exposed to a high day and night temperature for 204 h is shown on the left bottom side of the panel. By clicking on the spectrum button, individual pixel spectra of several pixels are plotted next to the image. On the rightmost bottom panel, the mean spectrum is plotted. The right top panel displays measurements of spatial–spectral phenotype characteristics of the seed. Furthermore, the spectrum button in Figure 4 enables the user to point the cursor at a pixel location to see the spectra of that pixel on the right side of this interface. The feature extraction button is used to calculate shape features of the seed image displayed in a panel in the GUI. In order to extract phenotype characteristics, the following features are extracted from the rice seed images [16].

### 2.3. Hyperspectral Seed Graphical User Interfaces

Four Graphical User Interfaces (GUIs) have been developed for the extraction of rice seed images and for feature extraction. Figure 3 shows the GUI for individual seed image extraction from a panel image containing several seeds. The user can input the path where the images have to be saved, as well as the category or class and subclass. The button for feature extraction is shown in the GUI in Figure 4. The features are described below:

If the boundary of the rice seed is represented by a polygonal curve, the *diameter* of a boundary is defined by

(2) $$diameter\left(B\right)=\max [D\left(p_{i},p_{j}\right)]$$

where D is a distance measure and $p_{i}$ and $p_{j}$ are points on the boundary. The value of the *diameter* and the orientation of a line segment connecting the two extreme points that comprise the *diameter* is called the major axis. The line perpendicular to the major axis is called the minor axis. The ratio of the major axis to the minor axis is called the *eccentricity* given by

(3) $$eccentricity=\sqrt{1-{\left(b/a\right)}^{2}},\;a\geq b\;$$

where *a* is half the length of the major axis and *b* is half the length of the minor axis. The surface *area* of the rice seed is the *area* of an ellipse given by

(4) area=πab 

The perimeter of an ellipse is given by

(5) $$p=\pi \left(a+b\right)\;$$

A more frequently used feature descriptor for shape is *compactness* given by

(6) $$compactness=p^{2}/A\;$$

where *A* is the *area*. The *roundness* or circularity is a dimensionless measure given by

(7) $$roundness=\frac{4\pi A}{p^{2}}\;$$

The mean intensity of the pixels in the seeds is given by

(8) $$X\;=\;\frac{1}{N}\sum_{i=1}^{N}x_{i}\;$$

**Figure 5:**
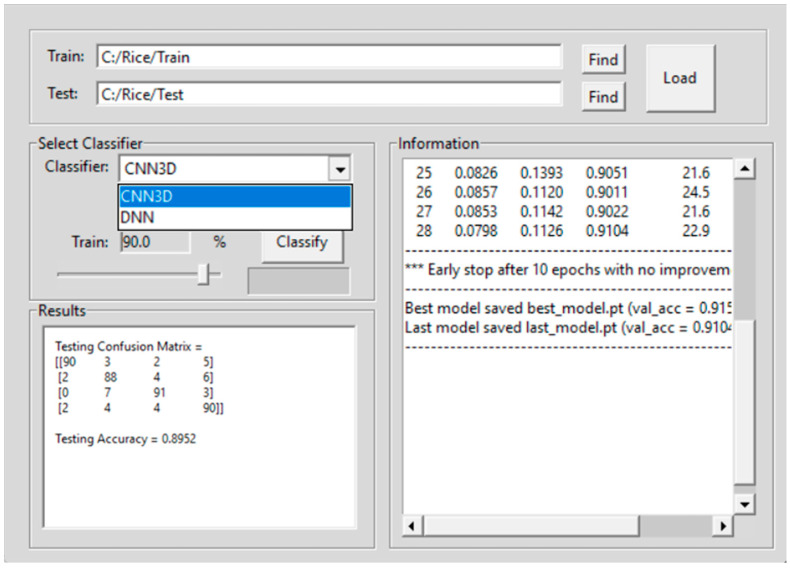
Graphical user interface for seed image classification using 3D-CNN and DNN.

Some of these features are shown in the top panel of the GUI in Figure 4. The selection of the classification button opens a fifth GUI shown in Figure 5. This GUI implements two DL methods for seed image classification. The first is the 3D-CNN which is an image-based classification method and the second is a DNN which is a pixel based classification method.

Figure 5 shows the GUI for loading the training HSI rice seed images from the train folder, and for loading the testing HSI rice seed images from the test folder. The link to download the codes for the GUI is provided in the Supplementary Materials.

### 2.4. 3D-Convolutional Neural Network

The architecture of a 3D-CNN consists of 3D convolutional layers, 3D pooling layers, and fully connected layers. Each convolutional layer in a 3D-CNN applies multiple 3D filters to the input data. The result of each convolution is passed through a non-linear activation ReLu function to introduce non-linearities into the model. The resulting data is then subjected to 3D pooling, using a 3D pooling operation to reduce the dimensionality of the feature volume. Finally, the output of the last pooling layer is fed into one or more fully connected layers to produce the final output of the model.

3D convolutions are performed similar to 2D convolutions in a CNN. However, in a 3D-CNN, the convolutional kernel has three dimensions instead of two. We have a 3D input I of size C×D×H×W, where *C* is the number of channels, *D* is the depth of the image, *H* is the height of the image, and *W* is the width of the image. To perform a 3D convolution on *I*, a 3D kernel *K* of size $C^{\prime }\times D^{\prime }\times H^{\prime }\times W^{\prime }$ is used, where *C*’ is the number of output channels, *D*’ is the depth of the kernel, *H*’ is the height of the kernel, and *W*’ is the width of the kernel. The 3D convolution operation is defined as the following:

(9) $$O_{i,j,k}=\sum_{d=0}^{D^{\prime }-1}\sum_{h=0}^{H^{\prime }-1}\sum_{w=0}^{W^{\prime }-1}\sum_{c=0}^{C-1}\sum_{c^{\prime }=0}^{C^{\prime }-1}I_{i+d,\;\;j+h,k+w,c}K_{d,h,w,c,c^{\prime }}$$

where *O* is the output of the convolution at position (*i*, *j*, *k*), *I* is the input volume, *K* is the kernel volume, *D*’ is the depth of the kernel, *H*’ is the height of the kernel, *W*’ is the width of the kernel, *C* is the number of input channels, *C*’ is the number of output channels, and *d*, *h*, *w*, *c*, and *c*’ are indices that iterate over the kernel and input dimensions. The 3D pooling operation is performed similarly to the 2D pooling operation in a CNN. The goal of 3D pooling is to reduce the dimensionality of the feature volume by subsampling the features in all three dimensions.

### 2.5. 3D-CNN Training and Validations

**Figure 6:**
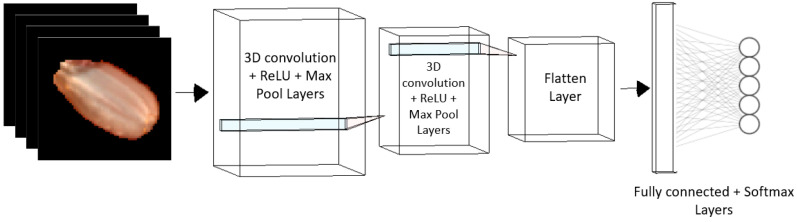
3D-CNN rice seed classification architecture.

Eighty percent of images are used for training the 3D-CNN, ten percent for validation, and ten percent for testing. The whole hypercube image of each seed is used to train the network. Since the imaging systems acquire lesser images, data augmentation is used to generate more images for training the 3D-CNN architecture. With data augmentation, the total number of images used for each class is 40 images. The workflow for the 3D-CNN-based classification is shown in Figure 6. The architecture consists of a 3D convolution layer with the Rectified Linear Unit (ReLu) and Max Pool operations. The output from the second 3D convolution layer is flattened and input to a fully connected layer with Softmax operation. The output of this layer is the classification of the seed image into one of the trained categories.

### 2.6. Deep Neural Network

The input of the DNN are the pixel vectors *X* of size 268 corresponding to the number of bands. The pixels are arranged in a matrix of dimension (numbers of pixels x numbers of bands). Each row of pixels is input to the DNN with its corresponding initial weights. The following notation is used for the DNN:

(10) $$\mathrm{Data}=X_{ij}^{l}$$

(11) $$\mathrm{Weights}=W_{ij}^{l}$$

where *i* corresponds to the index pixel (row), *j* corresponds to the values of the vector pixel and *l* corresponds to the layer. 

The process that occurs in each hidden layer is defined by the following operation. Taking for example the first pixel (neuron) in the first hidden layer:

(12) $$\sum =\left(X_{11}^{1}*W_{11}^{1}\right)+\left(X_{12}^{1}*W_{12}^{1}\right)+.\;.\;.+\left(X_{1n}^{1}*W_{1n}^{1}\right)$$

In vector form, it is the following:

(13) $$\sum =X_{1}^{1}.W_{1}^{1}$$

where

(14) $$\;X_{1}^{1}= [X_{11}^{1},X_{12}^{1},\ldots ,X_{1n}^{1}]\;\mathrm{and}\;W_{1}^{1}= [W_{11}^{1},W_{12}^{1},\ldots ,W_{1n}^{1}]\;$$

Adding a bias vector, the following general equation for the DNN is obtained:

(15) Z=X.W+b

where *b* is the bias tensor. 

The next step is to pass *Z* through a non-linear activation function. For the DNN proposed in this work, the Rectified Linear Unit activation function (ReLu) is used:

(16) $$y=\mathrm{ReLu}\left(Z\right)\;$$

Once the DNN is defined, we proceed to the training process using a lost function which is categorical cross entropy and the optimizer is the Adam optimizer. 

### 2.7. DNN Training and Validation

**Figure 7:**
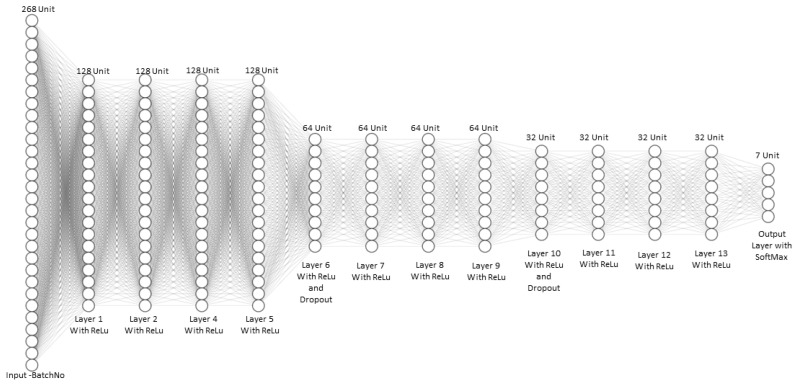
DNN rice seed classification architecture.

The architecture for the pixel-based classification scheme using DNN is given in Figure 7. The input data that is provided to the DNN at the input layer is the pixel spectral vector; in this case, each pixel vector has a dimension of 268 (number of band). The DNN consists of one input layer, 12 hidden layers, and finally an output layer using Softmax operation as shown in Figure 7. For the classification of rice images using DNN, 70% of the pixels of each rice seed from the treatments is used to train the model. The remaining 30% of the pixels is used for validation. Both DL methods are used for classifying two types of classification experiments. The first one is the classification of rice seeds or pixels of rice seeds. The results are evaluated using the Average Accuracy (AA), Overall Accuracy (OA), and Kappa score for the 3D-CNN, and the precision, recall, and f1-score metrics for the DNN. These performance metrics are shown in Table 2 In Table 2, TP stands for true positive, FP for false positive, TN for true negative, and FN for false negative. P and N are the total number of positive and negative samples for each class, respectively. The Kappa score tests the inter-reliability of the results, i.e., how much of the accuracy is obtained by chance. Po is the proportion of observed agreement, and Pe is the proportion of agreement expected by chance. Precision score measures the proportion of true positive samples among all samples that have been predicted as positive by the classifier. Precision is a useful metric to assess the importance of classification models and is used with recall and f1-score. Recall is the proportion of true positive samples among all actual positive samples in the dataset. A higher recall indicates that the model is better at identifying positive samples. A higher f1-score indicates better overall performance, considering both precision and recall. 

The Python codes for the DL framework for hyperspectral seed image calibration, preprocessing, segmentation, and DL neural network classification are made in a Github link provided in the Supplementary Materials. 

## 3. Results

This section presents the results using the 3D-CNN architecture and the DNN architecture for classification. About 30 images from each category of rice seed HSI are used in the training, testing, and validation phases of the 3D-CNN architecture. All the rice seed images are extracted from a panel of seeds using the GUI presented in Figure 2 and Figure 3. The GUI in Figure 4 is used to examine the spectra of the HSI seed images. GUI in Figure 5 is used to upload the training and testing images for each experiment. Two types of experiments are conducted. The first is to classify the rice seeds from the five different treatments for each hour of exposure. The second is to classify the rice seeds for different hours of exposure under each treatment class. The classification results of the first experiment are given in Table 3, and the classification results for the second experiment are given in Table 4, respectively. 

**Figure 8:**
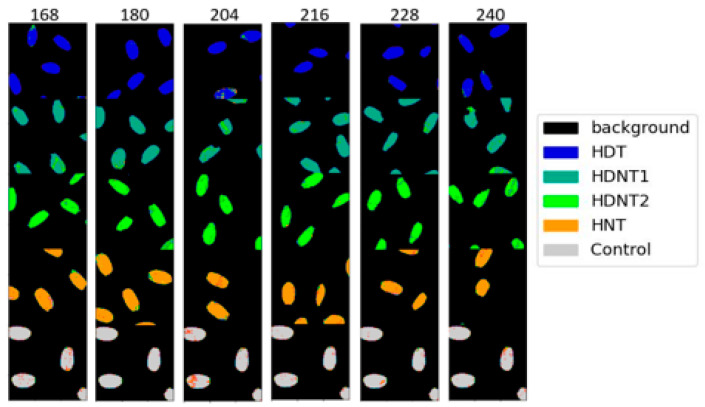
Classification map obtained from DNN classification of HSI of rice seeds for different high day/night temperature treatments.

**Figure 9:**
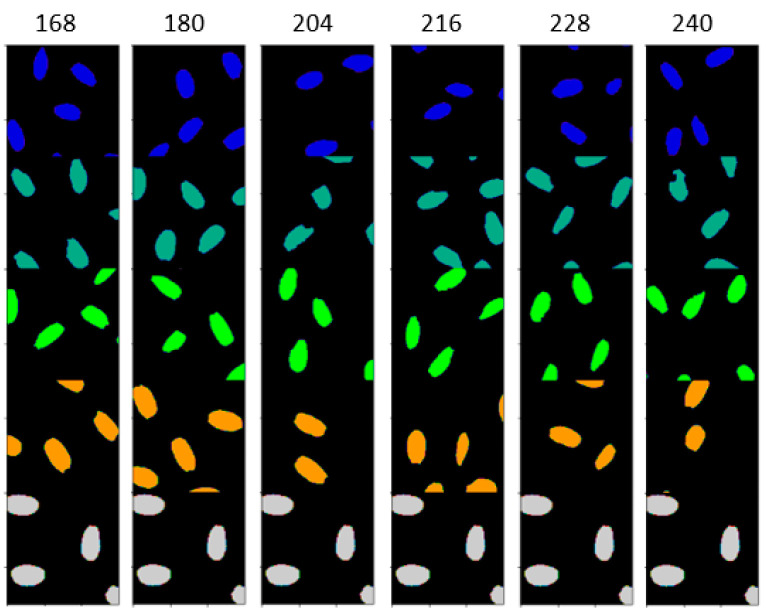
Ground truth for different high day/night temperature treatment.

**Figure 10:**
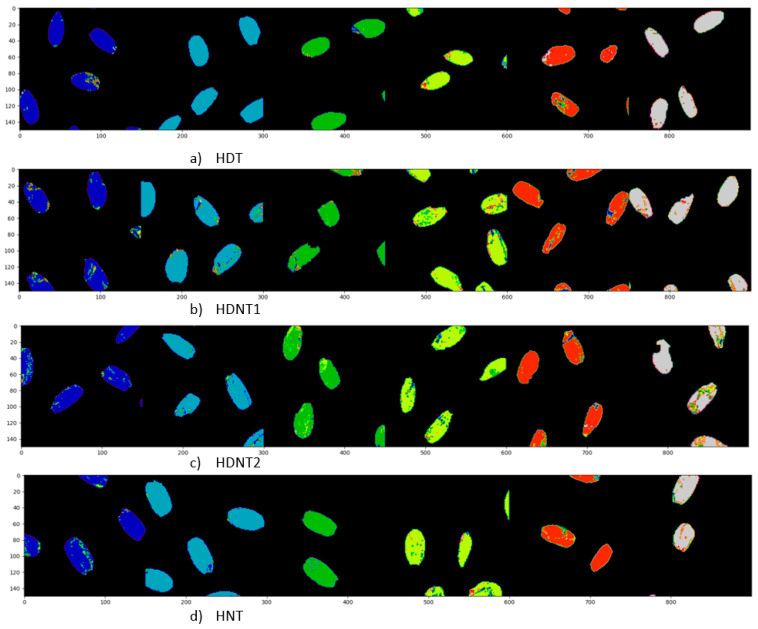
Classification map obtained from DNN classification of HSI of rice seeds for different exposure times for each treatment.

**Figure 11:**
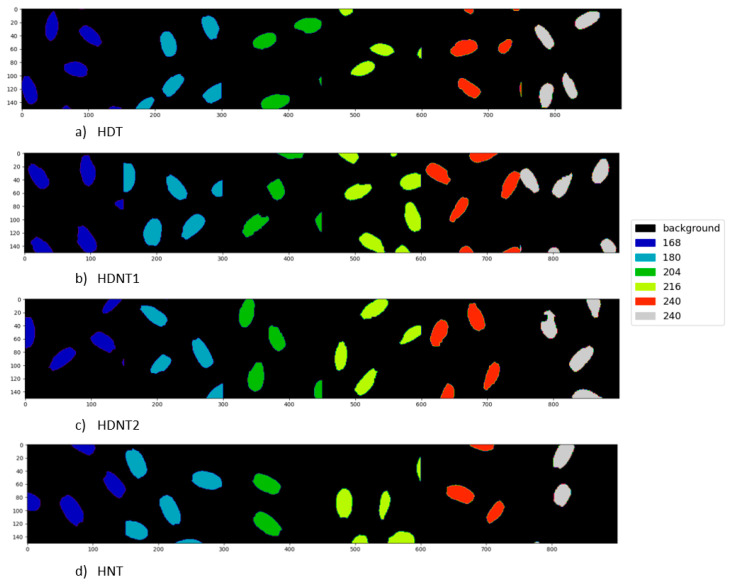
Ground truth for different exposure times for each high day/night temperature treatment classes.

Table 5 shows the classification results for the different temperature treatments of high day/night temperature for each exposure time using the DNN architecture. The classification maps for the different temperature treatments (classes) for each exposure time are shown in Figure 8, and the ground truths are shown in Figure 9, respectively. Table 6 gives the classification accuracies using the DNN for six different exposure times for each high day/night temperature treatment class. Figure 10 gives the classification map obtained from the DNN architecture for different exposure times, and Figure 11 gives the ground truth for this experiment, respectively.

## 4. Discussion

The accuracies are ordered in rows for the 3D-CNN in Table 3 and Table 4, and the accuracies are ordered in columns in the case of DNN in Table 5 and Table 6. For the DNN architecture the precision, recall, and f1-scores are also calculated, and hence they had to be ordered in columns. The average overall classification accuracy from Table 3 for classification of seeds from different temperature treatments using the 3D-CNN architecture is 91.33% which shows that the 3D-CNN performs well in discriminating between the seeds from different treatments for each exposure time. The average overall classification accuracy from Table 4 for different exposure times of rice seeds for all the treatments is 89.50%. 

The seeds from HDNT1 and HDNT2 treatments have lower accuracies for different exposure times. The average accuracy from Table 5 for different temperature treatments of rice seeds using the DNN architecture is 94.3%, which shows that the DNN performs well in discriminating between the seeds from different treatments for each exposure time. The precision scores are interpreted along with recall, which from Table 5 and Table 6 range between 0.86 and 1.0, showing that the classification model is good at identifying positive samples, and at the same time performs well in not identifying samples wrongly from another class; in other words, it has low false-positive errors. The f1-score is a combination of precision and recall, and ranges between 0.86 and 1.0, which shows a better overall performance of the DNN model. The lowest macro average and weighted average of precision, recall, and f1-score obtained are for the exposure times of 204 and 240 h. For 204 h of exposure time, the HDNT1 obtained the lowest accuracy values and for 240 h of exposure time, the treatments HDNT1 and HDNT2 obtained the lowest accuracy. Observing Figure 10, it can be seen that there are misclassifications in the rice seeds of the HDNT2 class into HDNT1 class and vice versa, which means that for these two exposure times these rice classes have similar pixel spectral characteristics. 

**Figure 12:**
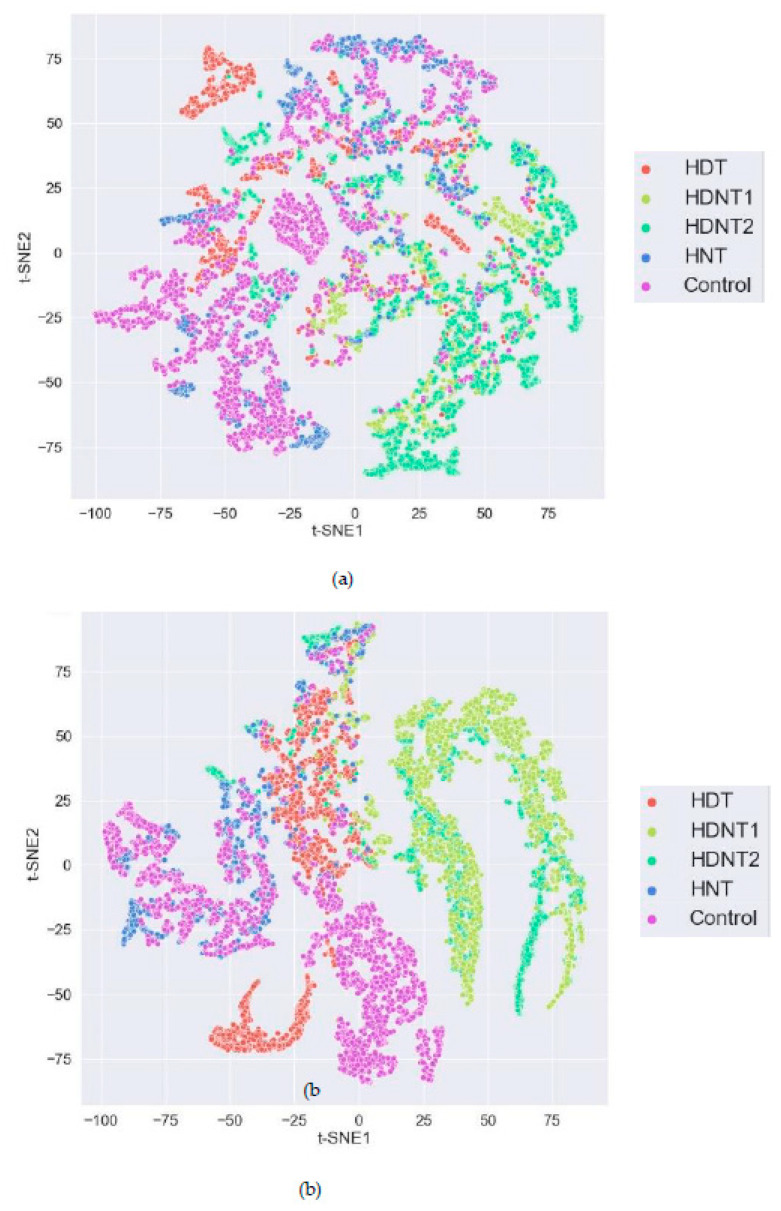
t-SNE visualization using two bands for the rice seed HSI from all treatments for (**a**) 204 h and (**b**) 240 h of high temperature exposure durations.

A t-Stochastic Nearest Neighbor Embedding (t-SNE) of the rice seed hyperspectral vectors have been performed and displayed in Figure 12. The t-SNE plots are made after taking the first 50 bands of the Principal Components Analysis (PCA) decomposition and performing a t-SNE visualization using the t-SNE1 (x-axis) and t-SNE2 (y-axis) components. These plots show that HSI of rice seeds grown under high temperatures have useful information in the different bands for distinguishing between the seeds grown under different hours of exposure to high temperatures. Most of the seed hyperspectral vectors from the five high temperature treatments are uncorrelated with each other as seen by the clustering of the colors. Hence, imaging spectroscopy is a promising tool for studying the impact of warming temperatures in the planet where seed crops are grown, and to study the phenotypic spatial–spectral changes in the seeds due to increase in day and night temperatures. 

In Figure 12, the light green points are the HDNT1 pixels and dark green points are the HDNT2 rice seed pixels, respectively, which show some correlation. These are the two classes of high temperature grown seeds that have similar values in spectral bands obtaining a lower accuracy compared to the other high temperature grown rice seeds. The classification accuracies using the DNN are 1% to 4% higher than that of the 3D-CNN, because the DNN performs a pixel-based classification, and there are more pixel samples to train the DNN. The 3D-CNN uses whole seed images for training the network. Whole seed HSI acquisition is costly and time consuming. However, the 3D-CNN also has performed well in distinguishing between the rice seeds from different temperature treatments and high temperature exposure durations.

### Comparison with State-of-the-Art Rice Seed HSI Classification

There are many publications on regular PNG or RGB rice seed variety classifications in the literature using machine learning and deep learning methods [17,18,19,20]. Table 7 summarizes the rice seed HSI classification. The work by Gao et al. [14] is the only one that classifies HSI rice seeds grown under high temperatures using 3D-CNN. Moreover, they classify only one temperature class against control totaling only two categories. Whereas, in this paper we have used 3D-CNN to classify rice seed HSI from four different temperature classes against the control and also to distinguish seeds exposed to different durations of high temperatures. 

There are also no studies on the State-Of-the-Art (SOA) on DNN for rice seed HSI pixel-based classification. The methods listed in Table 7 mostly use CNN but not 3D-CNN. The 3D-CNN architectures, as well as the DNN architecture presented in this framework, are able to perform a seed-based as well as a pixel-based classification of rice seeds grown under high day and/or night temperatures, obtaining higher accuracies than the SOA with a higher number of temperature treatment classes.

The DL framework for the processing and classification of rice seeds grown under high temperature can be extended to other varieties of rice seeds and grains to better understand the effects of warming temperatures on seed crops. The spectral phenotypic changes can be correlated with gene studies as well as evaluate the nutrition content of the seeds grown under higher temperatures. The DL software application runs without the installation of Python software, and can be used for calibration, processing, segmentation, feature extraction, and classification of other types of hyperspectral images of seeds such as wheat, maize, corn, watermelon, beet, and soybean.

## 5. Conclusions

A DL framework for the calibration, preprocessing, segmentation, and classification of rice seed hyperspectral images has been presented. The 3D-CNN architecture performs well in classifying the rice seeds from different temperature treatments, as well as classifying the seeds from subclasses of six temperature exposure durations. The DNN architecture uses the spectral information in each pixel of the rice seed images and performs a pixel-based classification obtaining a higher accuracy for all the four temperature treatments as well as for sub classes of varying temperature exposure durations. The rice seed hyperspectral images from the highest day and night temperature of 36/32 degree Celsius gives the lowest accuracy, showing that higher temperatures alter the seed spectral–spatial characteristics drastically making it non-discriminable from seeds from the other treatment classes. The HSI and DL methods presented here can be applied in precision agriculture to study the impact of environmental variables on grain varieties.

## Figures and Tables

**Table 1:** Temperature treatments classes of rice seeds.

Treatment Class	Day/Night Temperature °C	Number of Images
Control	28/23	40
High day/night temperature 1 (HDNT1)	36/32	40
High night temperature (HNT)	28/28	40
High day temperature (HDT)	36/23	40
High day/night temperature (HDNT2)	36/28	40

**Table 2:** Performance metrics used for 3D-CNN and DNN.

Performance Metrics	Formula
Precision	$\frac{TP}{TP+FP}$
Recall	$\frac{TP}{TP+FN}$
F1-Score	$2*\frac{precision*Recall}{Precision+Recal}$
Average Accuracy (AA)	$\frac{TP+TN}{TP+TN+FP+FN}$
Overall Accuracy (OA)	$\frac{TP+TN}{\left(P+N\right)}$
Kappa (K)	$\frac{Po-Pe}{1-Pe}$

**Table 3:** Classification accuracy for different treatments of high day and night temperatures.

	High Temperature Treatment Classes							
Exposure	HDT	HDNT1	HDNT2	HNT	Control	AA	OA	K
168	0.93	0.91	0.93	0.93	0.92	0.92	0.91	0.91
180	0.91	0.9	0.89	0.92	0.89	0.91	0.9	0.89
204	0.94	0.93	0.93	0.94	0.94	0.94	0.94	0.94
216	0.92	0.91	0.91	0.9	0.93	0.92	0.92	0.91
228	0.91	0.91	0.9	0.91	0.91	0.91	0.9	0.91
240	0.92	0.92	0.92	0.93	0.92	0.92	0.91	0.92

**Table 4:** Classification accuracy for different exposure times to high temperatures (hours of exposure).

	High Temperature Exposure Time (Hours)								
Classes	168	180	204	216	228	240	AA	OA	K
HDNT1	0.89	0.88	0.88	0.89	0.89	0.89	0.89	0.89	0.88
HDNT2	0.91	0.9	0.92	0.9	0.89	0.9	0.9	0.89	0.88
HDT	0.91	0.92	0.92	0.9	0.91	0.9	0.91	0.91	0.9
HNT	0.91	0.92	0.92	0.9	0.91	0.91	0.91	0.9	0.89

**Table 5:** Classification results for different high day/night temperature treatments using the DNN architecture.

	168 h	180 h	204 h	216 h	228 h	240 h
Classes	Precision	Precision	Precision	Precision	Precision	Precision
HDT	0.97	1	0.92	1	0.99	0.98
HDNT1	0.92	0.96	0.85	0.96	0.97	0.86
HDNT2	0.95	0.9	0.93	0.91	0.96	0.86
HNT	0.93	0.96	0.95	0.96	0.92	0.96
Control	0.96	1	0.94	0.98	0.98	0.96
Macro average	0.95	0.96	0.92	0.96	0.96	0.92
Weighted average	0.95	0.97	0.92	0.96	0.97	0.92
Classes	recall	recall	recall	recall	recall	recall
HDT	0.97	1	0.98	0.96	1	1
HDNT1	0.97	0.91	0.86	0.96	0.95	0.86
HDNT2	0.88	0.95	0.89	0.95	0.96	0.84
HNT	0.95	0.99	0.88	0.95	0.96	0.9
Control	0.96	0.99	0.96	0.99	0.97	0.99
Macro average	0.94	0.97	0.92	0.96	0.97	0.92
Weighted average	0.95	0.97	0.92	0.96	0.97	0.92
Classes	f1-score	f1-score	f1-score	f1-score	f1-score	f1-score
HDT	0.97	1	0.95	0.98	1	0.99
HDNT1	0.94	0.94	0.85	0.96	0.96	0.86
HDNT2	0.91	0.92	0.91	0.93	0.96	0.85
HNT	0.94	0.97	0.92	0.96	0.94	0.93
Control	0.96	0.99	0.95	0.98	0.97	0.97
Accuracy	0.95	0.97	0.92	0.96	0.97	0.92
Macro average	0.94	0.96	0.91	0.96	0.97	0.92
Weighted average	0.95	0.97	0.92	0.96	0.97	0.92

**Table 6:** Classification results for different exposure times for each temperature treatment using the DNN architecture.

	HDT	HDNT1	HDNT2	HNT
Classes	Precision	Precision	Precision	Precision
168	0.95	0.89	0.91	0.91
180	0.99	0.91	0.93	0.98
204	0.96	0.86	0.81	0.94
216	0.86	0.83	0.86	0.89
228	0.86	0.9	0.9	0.92
240	0.98	0.94	0.78	0.91
Macro average	0.94	0.89	0.87	0.93
Weighted average	0.95	0.89	0.87	0.93
Classes	recall	recall	recall	recall
168	0.96	0.83	0.83	0.92
180	0.97	0.98	0.92	0.96
204	0.96	0.87	0.88	0.92
216	0.99	0.92	0.76	0.89
228	0.9	0.82	0.9	0.96
240	0.91	0.89	0.93	0.91
Macro average	0.95	0.89	0.87	0.93
Weighted average	0.95	0.89	0.87	0.93
Classes	f1-score	f1-score	f1-score	f1-score
168	0.95	0.86	0.87	0.91
180	0.98	0.94	0.92	0.97
204	0.96	0.87	0.84	0.93
216	0.92	0.87	0.81	0.89
228	0.88	0.86	0.9	0.94
Accuracy	0.95	0.89	0.87	0.93
Macro average	0.94	0.89	0.87	0.93
Weighted average	0.95	0.89	0.87	0.93

**Table 7:** State-of-the-Art in rice seed HSI Classification.

Year	Authors	Algorithm	Number Classes	Overall Accuracy
2021	T. Gao et al. [14]	Rice seed HSI classification using 3D-CNN (high temperature)	2	97.5%
2021	T. Gao et al. [14]	Rice pixel based HSI classification (high temperature)	2	94.21%
2019	Z. Qui et al. [8]	Regular rice seed HSI image classification using CNN	4	87%
2018	I.Hatnuntawech et al. [11]	Regular rice seed HSI classification using ResNet-B	6	91.09%
	Proposed method	High temperature grown rice seed HSI using 3D-CNN	6	91.33%
	Proposed method	High temperature grown rice seed HSI using DNN	6	94.83%

## Supplementary Materials

The codes for the DL framework for hyperspectral seed image calibration, preprocessing, segmentation, and classification are available at: https://gitfront.io/r/vido6/vC64GLsxCDZx/classificationRice/, accessed on 23 March 2023.
